# Supplementary material for: Mechanisms governing the pioneering and redistribution capabilities of the non-classical pioneer PU.1
Source: Nat Commun. 2020 Jan 21;11:402. doi: 10.1038/s41467-019-13960-2 (PMC6972792; doi:10.1038/s41467-019-13960-2)
Supplement: Supplementary file 7 — Source data [file 41467_2019_13960_MOESM7_ESM.zip › Source_Data/Figure6/Figure6B_MotifScanOutput/FLI1_mutPU.1/homerResults/motif5.similar.html]

motif5

## Information for motif5

A
C
G
T
A
C
T
G
A
C
G
T
A
C
T
G
A
C
T
G
A
T
C
G
G
T
C
A
  
Reverse Opposite:  

C
A
G
T
A
T
G
C
A
G
T
C
A
G
T
C
C
G
T
A
A
G
T
C
C
G
T
A
  

|  |  |
| --- | --- |
| p-value: | 1e-208 |
| log p-value: | -4.790e+02 |
| Information Content per bp: | 1.903 |
| Number of Target Sequences with motif | 4309.0 |
| Percentage of Target Sequences with motif | 42.00% |
| Number of Background Sequences with motif | 9915.0 |
| Percentage of Background Sequences with motif | 26.01% |
| Average Position of motif in Targets | 101.3 +/- 53.5bp |
| Average Position of motif in Background | 99.8 +/- 61.8bp |
| Strand Bias (log2 ratio + to - strand density) | 0.0 |
| Multiplicity (# of sites on avg that occur together) | 1.29 |
| Motif File: | file (matrix) reverse opposite |

### Similar de novo motifs found

|  |  |  |  |  |  |  |  |
| --- | --- | --- | --- | --- | --- | --- | --- |
| Rank | Match Score | Redundant Motif | P-value | log P-value | % of Targets | % of Background | Motif file |
| 1 | 0.770 | C A G T T A C G G C A T C T A G A C T G C T A G A C T G A C T G G T C A C T A G C A T G | 1e-155 | -358.309458 | 29.72% | 17.44% | motif file (matrix) |
| 2 | 0.831 | A G T C C A G T G T A C A G T C A G T C G T C A A G T C C G T A A T G C | 1e-124 | -287.778680 | 27.96% | 17.12% | motif file (matrix) |
| 3 | 0.810 | A T C G G A T C C A G T C A G T T A C G A C G T A C T G C T A G A T C G C T G A C T A G | 1e-45 | -105.503853 | 5.12% | 2.27% | motif file (matrix) |
